# Keeping it simple: the value of an irreducibly simple climate model

**DOI:** 10.1007/s11434-015-0856-2

**Published:** 2015-08-06

**Authors:** Christopher Monckton of Brenchley, Willie W.-H. Soon, David R. Legates, William M. Briggs

**Affiliations:** Science and Public Policy Institute, Haymarket, VA 20169 USA; Harvard-Smithsonian Center for Astrophysics, Cambridge, MA 02138 USA; Department of Geography, University of Delaware, Newark, DE 19716 USA; New York, NY 10021 USA

**Keywords:** Climate change, Climate sensitivity, Climate models, Global warming, Temperature feedbacks

## Abstract

Richardson et al. (Sci Bull, 2015. doi:10.1007/s11434-015-0806-z) suggest that the irreducibly simple climate model described in Monckton of Brenchley et al. (Sci Bull 60:122–135, 2015. doi:10.1007/s11434-014-0699-2) was not validated against observations, relying instead on synthetic test data based on underestimated global warming, illogical parameter choice and near-instantaneous response at odds with ocean warming and other observations. However, the simple model, informed by its authors’ choice of parameters, usually hindcasts observed temperature change more closely than the general-circulation models, and finds high climate sensitivity implausible. With IPCC’s choice of parameters, the model is further validated in that it duly replicates IPCC’s sensitivity interval. Also, fast climate system response is consistent with near-zero or net-negative temperature feedback. Given the large uncertainties in the initial conditions and evolutionary processes determinative of climate sensitivity, subject to obvious caveats a simple sensitivity-focused model need not, and the present model does not, exhibit significantly less predictive skill than the general-circulation models.

**Electronic supplementary material:**

The online version of this article (doi:10.1007/s11434-015-0856-2) contains supplementary material, which is available to authorized users.

## Introduction

### Defects in complex models’ output

Outputs from the general-circulation models cited in IPCC’s five *Assessment Reports* FAR, SAR, TAR, AR4 and AR5 [1–5] were examined in [6] using a simple model calibrated against IPCC’s central climate-sensitivity estimates in AR4-5 and were found to overestimate observed air temperature trends. Reasons for this hot running were discussed. The simple model, using parameter values consistent with a growing body of papers [e.g., 7–33] that report climate sensitivity to be below current central estimates, showed that in at least five significant respects the general-circulation models’ approach was questionable:

1. The assumption that temperature feedbacks will double or triple direct warming is unsound. Feedbacks may well reduce warming, not amplify it (see, e.g., [16, 19]).
2. The Bode system-gain equation models mutual amplification of feedbacks in electronic circuits, but, when models erroneously apply it to the essentially thermostatic climate on the assumption of strongly net-amplifying feedbacks, its use leads to substantial overestimation of global warming.
3. Climate modelers have failed to cut their central estimate of global warming in line with a new, lower estimate of the feedback sum (AR5, fig. 9.43). They still predict 3.3 K warming per CO_2_ doubling, when on this ground alone they should only predict 2.2 K, of which direct warming and feedbacks each contribute about 50 %.
4. Though general-circulation models suggest 0.6 K man-made warming is “in the pipeline” even if CO_2_ emissions cease, the simple model, supported by almost two decades without significant global warming [34], suggests there is no committed but unrealized man-made warming still to come.
5. AR5’s extreme RCP 8.5 forcing scenario predicting ~12 K anthropogenic warming is unjustifiable. It was based on CO_2_ concentration growing at 5.5 ppmv year^−1^ this century, though AR4’s central estimate was below 3.5 ppmv year^−1^ and the current growth rate [35] is little more than 2 ppmv year^−1^.

In [6], it was concluded that once due allowance is made for these and other shortcomings in the general-circulation models, the likely global warming in response to a doubling of CO_2_ concentration is not 3.3 K but 1 K or less and that even if all available fossil fuels are combusted <2.2 K warming will result.

### The simple model

The irreducibly simple model presented in [6], encapsulated in (1), determines the surface temperature response Δ*T*_*t*_ to anthropogenic radiative forcings and consequent temperature feedbacks over any given period of years *t*:

1 $$ \begin{aligned}\Delta T_{t} & = q_{t}^{ - 1}\Delta F_{t}  r_{t}  \lambda_{\infty } \\ & = q_{t}^{ - 1} k\; { \ln }\left( {\frac{{C_{t} }}{{C_{0} }}} \right)r_{t}  \lambda_{\infty } \\ & = q_{t}^{ - 1} k\; { \ln }\left( {\frac{{C_{t} }}{{C_{0} }}} \right)r_{t}  \lambda_{0}  G \\ & = q_{t}^{ - 1} k \;{ \ln }\left( {\frac{{C_{t} }}{{C_{0} }}} \right)r_{t}  \lambda_{0 } \left( {1 - g} \right)^{ - 1} \\ & = q_{t}^{ - 1} k\; { \ln }\left( {\frac{{C_{t} }}{{C_{0} }}} \right)r_{t}  \lambda_{0} \left( {1 - \lambda_{0} f_{t} } \right)^{ - 1} , \\ \end{aligned} $$

where $q_{t}$ is the fraction of total anthropogenic forcing represented by CO_2_ over *t* years, and its reciprocal allows for non-CO_2_ forcings as well as the CO_2_ forcing; Δ*F*_*t*_ is the radiative forcing in response to a change in atmospheric CO_2_ concentration over *t* years, which is the product of a constant *k* and the proportionate change (*C*_*t*_ / *C*_0_) in CO_2_ concentration over the period, where *C*_0_ is the unperturbed concentration ([36]; TAR, ch. 6.1); *r*_*t*_ is the transience fraction, which is the fraction of equilibrium sensitivity expected to be attained over *t* years, allowing any delayed-response profile to be modeled as a time series; *λ*_∞_ is the equilibrium climate-sensitivity parameter, which is the product of the Planck or zero-feedback sensitivity parameter *λ*_0_ (AR4, p. 631 fn.) and the open-loop or system gain *G*, which is itself the reciprocal of 1 minus the closed-loop gain* g*, which is in turn the product of *λ*_0_ and the sum *f*_*t*_ of all temperature feedbacks acting over the period.

In [6], the simple model encapsulated in (1) is described thus: “This simple equation represents, in an elementary but revealing fashion, the essential determinants of the temperature response to any anthropogenic radiative perturbation of the climate, and permits even the non-specialist to generate respectable approximate estimates of temperature response over time. It is not, of course, intended to replace the far more complex general-circulation models: rather, it is intended to illuminate them.”

## Criticisms of the simple model

In [37], various criticisms of the simple model in [6] were tendered. We now consider those criticisms *seriatim*.

### Form of the simple model

It is stated in [37] that the simple model’s “extreme simplification necessarily leaves out many physical processes”. The model was intentionally simple. The aim was to allow even an undergraduate student of climatological physics to understand the key forcings, feedbacks and other parameters determinative of climate sensitivity and to generate respectable climate-sensitivity estimates that would serve to illuminate the outputs of the general-circulation models. General-circulation models are inherently complex, so that processes and feedbacks are often obscured. The goal of the simple model is to be transparent and to allow for direct evaluation of the response function associated with its key components. Indeed, so large are the uncertainties in the initial conditions and in the time-dependent processes by which the climate object evolves that the penalty in predictive skill arising from simplicity is smaller than might otherwise be the case. Every model is a simplification, and every simplification is an analogy, and every analogy breaks down at some point. Accordingly, [6] contained caveats about the limitations on the competence of a simple model.

### Focus on anthropogenic forcings

It is said in [37] that “the standard approach” that includes natural as well as anthropogenic forcings “is more useful” than the simple model “because both Δ*T*_*t*_ and Δ*F*_*t*_ may be estimated from observations”. However, attribution as between anthropogenic and natural temperature change is not trivial. Also, uniform near-global-temperature measurements only became available in 1979 with the two satellite lower-troposphere datasets. Uncertainties before that time do not provide a secure basis for attempting to determine climate sensitivity. Also, as [6] explained, the process of determining forcings and distinguishing them from feedbacks is subject to very large uncertainties. Even the usual central estimate of the CO_2_ forcing was reduced by 15 % on the basis of intercomparison between three models in [36]. In any event, the user of the simple model may incorporate terms for natural forcings where that is thought desirable.

### The transience fraction

It is argued in [37] that the transience fraction *r*_*t*_ should have been determined not by reference to a single pulse of forcing but by a convolution of the forcing series with the time-response function. Naturally, one could have presented a more complex model. However, the effect of assuming a single pulse rather than a time series of forcings is merely to increase the value of *r*_*t*_ somewhat, for the sake of caution, where *t* is sufficiently distant both from zero and from equilibrium. At equilibrium, the primary focus of [6], *r*_*t*_ is by definition unity. Likewise, if the feedback sum *f*_*t*_ is below 0.1, no large error arises from assuming *r*_*t*_ = 1. And it is made clear in [6] that the user is free to adopt any chosen time-series array of values for *r*_*t*_. Finally, the authors of [37] have not performed the calculation they had themselves recommended.

### Calibration of the simple model

It is stated in [37] that the projections of the simple model were not compared with observations. However, as explained in [6] and confirmed in [37], the model was calibrated against the central climate-sensitivity projections in AR4-5 and was found to reproduce those projections. In [6], parameter values somewhat different from IPCC’s values were then selected, assuming net-negative feedbacks and also assuming a transience fraction *r*_*t*_ = 1. The model was then run, determining a climate sensitivity of 1.0 [0.8, 1.3] K per CO_2_ doubling, in response to a CO_2_ radiative forcing 5.35 ln(2) = 3.7 W m^−2^.

The parameter values that generated this climate-sensitivity interval had not been fitted to past observations, since the intention was to base them purely on objective, theoretical considerations. Using those parameters, and with the aim of responding constructively to the suggestion that comparison with observation would be of value, the model has now been tested against observation. Some preliminary considerations should be borne in mind.

First, the still problematic tendency of the global-temperature datasets to overestimate the amplitude of anthropogenic warming over the past 150–250 years owing to contributions from non-climatic factors [38, 39] has been largely set to one side in the analysis that follows.

**Fig. 1:**
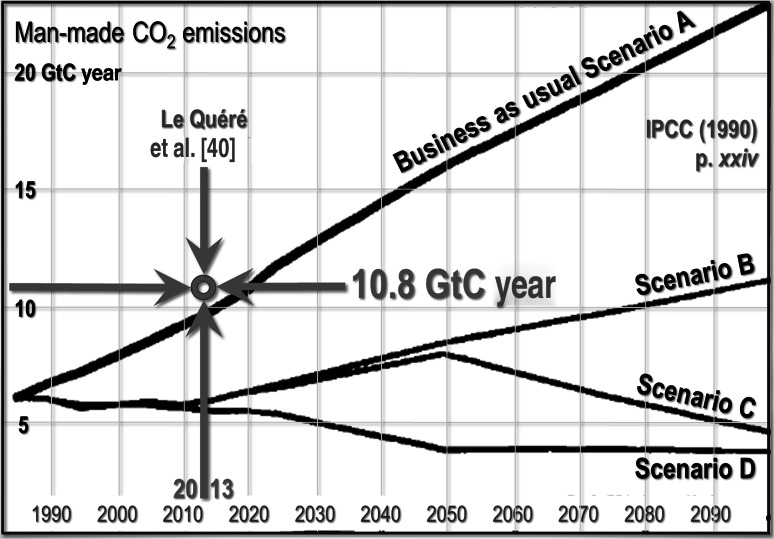
Predicted annual CO_2_ emissions (GtC) on four scenarios (FAR, p. *xxiv*) and 2012 outturn

Worse, we do not really know—perhaps even to within a factor 2—what is the magnitude of the total forcings since 1750. IPCC ([1], p. xxiv) provided a graph predicting future annual emissions of CO_2_ (Fig. 1). Scenario A, the “business-as-usual” case, predicted global emissions of almost 10 GtC year^−1^ by 2012. In [40], it was estimated that in 2013 global emissions were actually 10.8 GtC, somewhat above even the business-as-usual prediction in FAR. Scenario A, then, is closer than the other three FAR scenarios to the observed outturn.

**Fig. 2:**
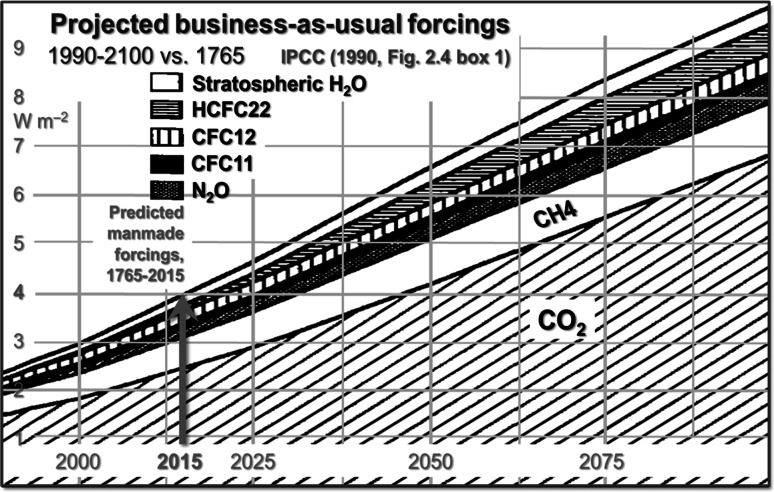
Projected business-as-usual radiative forcings, 1990–2100 versus 1765 (FAR, Fig. 2.4 box 1)

Why the factor-2 difference between the forcings of 4 W m^−2^ predicted in FAR (Fig. 2) and the estimated outturn of little more than half of that value, or 2.3 W m^−2^, in AR5 (Fig. SPM.5)? The chief reason is that from SAR onward IPCC introduced the notion that anthropogenic aerosol forcings were pronouncedly negative.

**Fig. 3:**
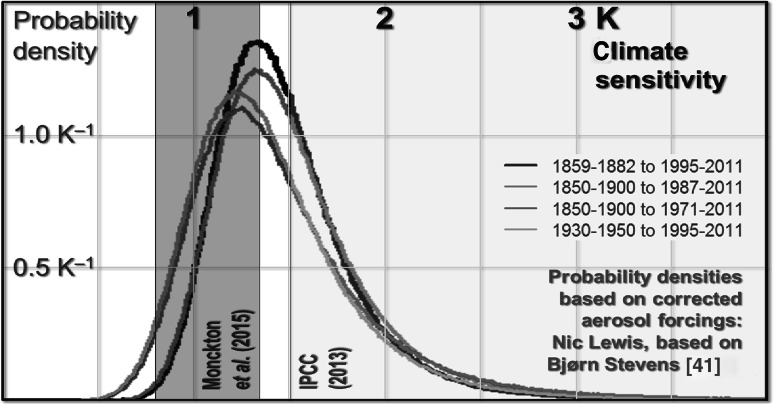
Probability densities based on four different aerosol reanalyses in [41], indicating climate sensitivity is most likely to fall in the region of 1.3 K. Equilibrium climate sensitivity intervals in [6] and in AR5 are indicated by dark and light shadings, respectively. Graph based on [42]

The literature, however, is divided on this subject. For instance, a recent paper [41] modeling climate sensitivity based on research showing that a strong aerosol forcing establishes itself early in the historical record finds sensitivity tightly constrained close to 1.3 K (Fig. 3). Bearing in mind these and other uncertainties, a number of observational test runs of the model were performed.

#### Observational test 1

One method of testing the simple model’s predictive skill is to compare its prediction of global warming in the 25 years 1990–2014 with those made by the IPCC on the business-as-usual Scenario A from 1990 to 2025 and also with observed outturn. The IPCC’s predictions (FAR, executive summary) were presented under the heading “How much confidence do we have in our predictions?” IPCC pointed out some uncertainties (clouds, oceans, etc.), but concluded:

Nevertheless … we have substantial confidence that models can predict at least the broad-scale features of climate change … There are similarities between results from the coupled models using simple representations of the ocean and those using more sophisticated descriptions, and our understanding of such differences as do occur gives us some confidence in the results.

FAR’s medium-term temperature-change prediction, “based on current models”, was “a likely increase in global mean temperature of about 1 °C above the present value by 2025” (p. *xii*). Later (p. *xxiv*), IPCC said, as shown in Fig. 8 on p. *xxii* of FAR, that the lower and upper bounds of its predictions were, respectively, 30 % below and 50 % above its best estimate. Accordingly, IPCC predicted warming of 1.0 [0.7, 1.5] K to 2025. Since the graph is close to linear from 1990 to 2025 and beyond, little error arises by assuming linear warming over the period, so that IPCC’s pro rata predicted warming interval from 1990 to 2014 was 0.71 [0.49, 1.06] K. However, observed warming over the quarter century from 1990 to 2014, taken as the linear trend on the RSS temperature dataset [43], was 0.26 K. Therefore, IPCC’s central prediction was more than 2.5 times observed outturn.

As for the simple model, taking the Planck sensitivity parameter *λ*_0_ = 0.31 K W^−1^ m^2^ and the transience fraction *r*_25_ = 1 (reflecting assumed net-negative feedback), adopting the values in [6, table 7] for the feedback sum *f* and the closed-loop gain *g,* and taking from FAR the CO_2_ forcing coefficient *k* = 6.3 and the CO_2_ fraction *q* = 0.7, the simple model predicts warming of 0.27 [0.23, 0.37] K since 1990, consistent with outturn (Table 1).

**Table 1:** Comparison of the 1990–2014 climate-sensitivity interval in the simple model with outturn taken as the RSS [43] linear trend and the intervals implicit in FAR on the business-as-usual emissions Scenario A, assuming that the proportionate change in CO_2_ concentration was 397/353 and that, as in FAR, the CO_2_ forcing coefficient *k* = 6.3 and the CO_2_ fraction *q* = 0.7

*f*	*g*	*λ*_∞_	Δ*F*_25_	Δ*T*_25_		
[6]	[6]	*λ*_0_(1 − *g*)^−1^	*q* ^−1^ *k* ln 397/353	Model *λ*_∞_ Δ*F*_25_	Obsv. [43]	FAR Scn.A
(W m^−2^ K^−1^)		(K W^−1^ m^2^)	(W m^−2^)	(K)	(K)	(K)
−1.60	−0.5	0.208		0.23		0.49
−0.64	−0.2	0.260	1.057	0.27	0.26	0.71
+0.32	+0.1	0.347		0.37		1.06

#### Observational tests 2–4

The simple model was also tested against IPCC’s estimates of net anthropogenic radiative forcings of 0.57 W m^−2^ from 1750 to 1950, 1.25 W m^−2^ from 1750 to 1980 and 2.29 W m^−2^ from 1750 to 2012 (AR5, Fig. SPM.5), under the assumptions that there was 0.1 K natural warming from 1750 to 1850, the year when HadCRUT4 [44] began (the central England record shows 0.1 K warming from 1750 to 1850) and that all warming since 1850 was anthropogenic. Under these assumptions, the simple model hindcasts anthropogenic warming of 0.15 [0.12, 0.20] K against 0.15 K observed from 1750 to 1950, 0.33 [0.26, 0.43] K against 0.28 K observed from 1750 to 1980 and 0.60 [0.48, 0.80] K against 0.66 K observed from 1750 to 2012 (Table 2).

**Table 2:** Modeled and observed global mean anthropogenic surface temperature anomalies based on anthropogenic forcings in AR5 (SPM.5), 1750–1950, 1750–1980 and 1750–2012

Period	*λ*_∞_	Δ*F*_anth_	Δ*T*_anth_	
AR5 SPM.5	*λ*_0_(1 − *g*)^−1^	AR5 SPM.5	Model *λ*_∞_ Δ*F*_anth_	Obsv. [44]
	(K W^−1^ m^2^)	(W m^−2^)	(K)	(K)
1750–1950	0.208		0.12	
	0.260	0.57	0.15	0.15
	0.347		0.20	
	0.208		0.26	
1750–1980	0.260	1.25	0.33	0.28
	0.347		0.43	
	0.208		0.48	
1750–2012	0.260	2.29	0.60	0.66
	0.347		0.80	

As in [37], no allowance was made in the four observational tests for the significant correlation between regional rates of economic growth and of climate warming in recent decades, suggesting that the urban heat-island effect has not been purged from the temperature datasets and may account for up to 0.2 K of the twentieth-century warming [45], or for the upward adjustment of some 0.3 K in recent observed terrestrial temperatures [46].

**Fig. 4:**
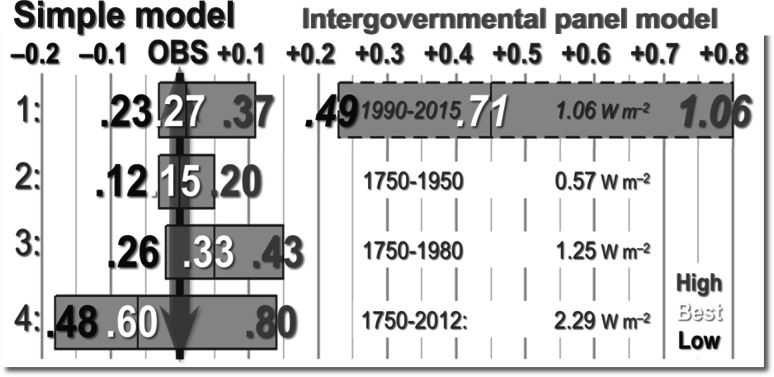
Four tests of the simple model’s hindcasts (solid-edged boxes central values in white) against observed global mean temperature anomalies from 1990 to 2015 (arrowed °C) and against (1) FAR’s near-term predictions (dashed edges) based on 1.00 [0.70, 1.50] K warming to 2025 and using AR5’s estimates of total anthropogenic forcings from 1750 to (2) 1950; (3) 1980 and (4) 2012. The observed HadCRUT4 [44] surface temperature anomalies (1) and RSS [43] satellite temperature anomalies (2–4) always fall on the simple model’s hindcast intervals (left); however, FAR’s near-term predictions (right) have proven to be much in excess of observation

Results of all four tests against observation are summarized in Fig. 4.

IPCC—on the advice of expert reviewers including the lead author of the present paper—has realized that the general-circulation models were exaggerating warming and has substituted its “expert assessment” for the models’ predictions, greatly reducing its medium-term warming projections in AR5 compared with FAR. In 1990, IPCC predicted medium-term warming at 0.3 [0.2, 0.4] K decade^−1^, near-halved in 2013, following observed warming at 0.14 K decade^−1^ since 1990, to just 0.17 [0.10, 0.23] K decade^−1^. Curiously, though, IPCC has not correspondingly reduced its equilibrium sensitivity interval [1.5, 4, 5] K, identical to the interval given 36 years ago in Jule Charney’s influential report [47] for the U.S. National Academy of Sciences.

### Further defects

In [37], it is argued that the interval of observations in [6], i.e., [0.0, 1.1] K century^−1^, the lower bound representing the RSS temperature change over the past 18 years 6 months, the upper bound representing the centennial equivalent of the warming since 1950 shown in the HadCRUT4 dataset, ought not to have been projected out to 2050. However, little turns on this graphical projection, which served merely to illustrate the very large divergence between the outturn to date and the models’ predictions, and to show how much wider that divergence will become if, as the simple model predicts, it persists to 2050. In particular, the statistical test that shows that the observed warming during one period differed from that found in [6] is likewise superfluous. The authors of [37] need have said no more than they used values, time points and temperature datasets that differed from those in [6].

The plots in Fig. 1 of [37] are not the result of out-of-sample predictions, but comprise model fits to the observed data. The plots are analogous to showing how close a regression line is over a scatter plot of already-observed data. All that these plots indicate is that over the chosen period and using the chosen temperature datasets, the CMIP5 outputs can be made to fit the data marginally better than those of the simple model. However, as the above four observational tests using both satellite and terrestrial datasets illustrate, the simple model outperforms the general-circulation models over the period since 1990 and is very close to observation at all other timescales.

The real test of a model is always predictive skill. That a model should fit observed data well is a necessary but not a sufficient criterion for predictive skill. In [37], the fallacy is perpetrated of assuming that better model fit implies better predictive skill. It does not, as is amply demonstrated in Figs. 6, 7, 8 and 9 here. Yet the observational tests described above show that the simple model fits observed data well. For the period since 1990, it outperforms the general-circulation models by a substantial margin.

Furthermore, RMSE model-fit statistics are generally a poor measure to use in validation because the in-sample model predictions are not univariate numbers, but “envelopes” that should be (but in [37] were not) treated probabilistically. A statistical technique such as the complete-rank probability score is far superior [48].

It is concluded in [37] that the simple model “may not be considered validated”. Here, the elementary error is made of assuming that a choice of parameter values that is thought inappropriate invalidates the model to which that choice of parameters was applied, rather than invalidating solely the chosen parameter values. The simple model, if informed with IPCC’s parameter values, generates climate sensitivities very close to those put forward by IPCC, as the authors of [37] themselves demonstrate, thereby—in their own terms—validating it. The simple model, if informed with the alternative parameter choices presented in [6], generates hindcasts remarkably close to observation, providing further validation.

What is more, the fact that the simple model was developed entirely from physical first principles and was not adjusted by regression to make it fit past temperature change is not a vice but a virtue. The fact that the model performs as well as it does without any need for such ex post facto adjustment is additional validation.

### Closed-loop gain

In [37], an incomplete summary is given of the grounds in [6] for considering it likely that the closed-loop feedback gain factor *g* < 0.1 (misdescribed in [37] as the “system gain”, which is the term usually applied—and applied in [6]—to the open-loop gain factor *G*). In fact, there is now extensive literature, some of it cited in [6], indicating on the basis of empirical evidence as well as theory that temperature feedbacks are not likely to be strongly net positive.

The parameters in [6] were chosen on the basis of the literature cited and on the basis of the inapplicability of the Bode system-gain equation to strongly net-positive feedbacks in the climate object. In [49], the relative characteristics of net-positive and net-negative feedbacks are described. The characteristics of the net-positive feedbacks are inconsistent with the thermostatic behavior of the climate, while those of net-negative feedback are consistent with it. In [50], it is demonstrated that climate feedbacks correspond neither to the concept of feedback used in control theory nor “in any literal sense to the concept of feedback as used in electronics … the figurative transfer of an amplification formula from another field into the climate area must not be seen as implying that some general physical principle is being invoked”. Yet IPCC (e.g., AR4, p. 631 fn.) uses the Bode system-gain relation without appropriate cautions as to its inapplicability where strongly net-positive feedbacks are posited. The authors of [37] are, of course, as free as anyone else to choose their own parameter values.

### Determination of the feedback sum from paleoclimate evidence

The authors of [37] cite [51] as contradicting the assertion in [6], following the cryostratigraphic temperature reconstruction in [52], that paleoclimate temperature varied by only 3.5 K either side of the mean over the past 810 ka. They say Zachos et al. [51] found that during the late Paleocene thermal maximum 55 Ma B.P. temperatures in high latitudes rose by 8 K, perhaps as a result of forcing from greenhouse-gas increases.

However, with respect, 810 ka is not the same interval as 55 Ma. Over the 54 Ma from the Paleocene–Eocene thermal maximum to the commencement of the currently available cryostratigraphic temperature reconstruction, there were substantial and very significant changes in tectonic and continental configurations inconsistent in fundamental respects with today’s climatic environment.

Furthermore, the quotation from [51] given in [37] is incomplete: The words “and lesser amounts toward the equator” were omitted. Here, an error is made that is common among those unfamiliar with the consequences of poleward advection, one of the many fundamental and often underappreciated non-radiative transfers in the climate object [53, 54]. Cryostratigraphy reconstructs polar temperatures; but advection from the tropics approximately doubles at the poles any change in mean global temperatures. By this polar amplification, the 7 K global glacial-to-interglacial interval of temperature change shown in [52] is equivalent to 14 K at the poles.

The 8 K polar change even during the extreme and rapid events of the late Paleocene thermal maximum is thus well within the interval implicit in the cryostratigraphic record presented by [52]. Furthermore, in [51] it is made quite plain that the cause of that thermal maximum, whose onset may have occurred over as little as 1,000 years, is unknown. It is not thought that CO_2_ had anything to do with it, though [51] canvasses the possibility of a substantial dissociation and subsequent oxidation of 2000–2600 Gt of CH_4_ from abyssal clathrates.

In [37], it is also stated that, if temperature feedbacks were negative, small initial forcings owing to the Milankovitch cycles would not be capable of triggering glacial–interglacial transitions. Yet two suggested possible causes of the late Paleocene thermal maximum, discussed in [51], are the large forcings from abrupt deep-sea warming resulting from sudden changes in ocean circulation [55, 56] and massive regional slope failure [57].

### Ocean heat content

**Fig. 5:**
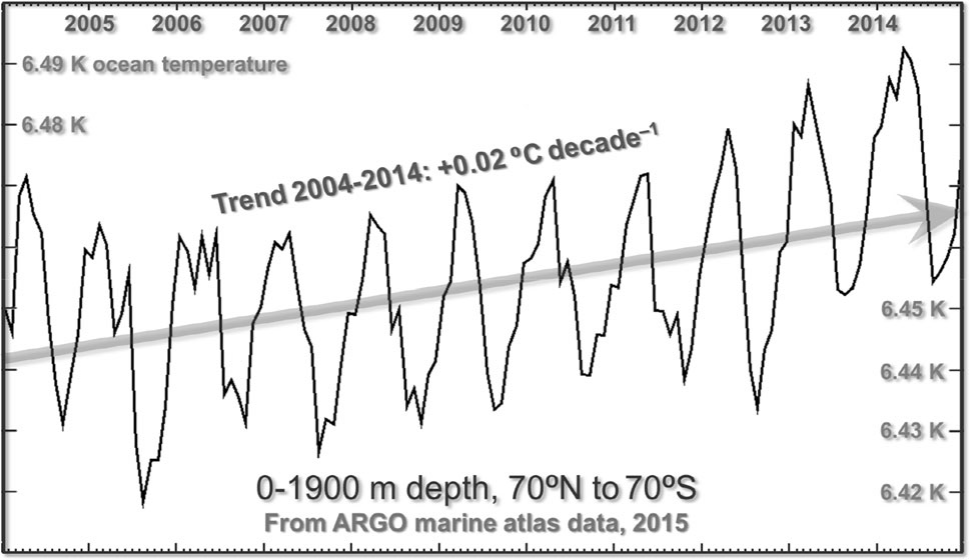
Ocean temperature, 0–1,900 m depth, 70°N–70°S, over the entire ARGO time series from 2004 to 2014. Since the ARGO buoys measure temperature, the ARGO data from 2004 to 2014 were obtained from the ARGO marine atlas and are shown together with the linear trend

In [37], it is asserted that the ARGO bathythermograph buoys found a “net heating” of about 0.5 W m^−2^ from 2000 to 2010. In fact, [58], cited in [37], inferred the energy imbalance from satellite observations and used ARGO measurements to determine whether there was an inconsistency between the indications from the two systems. Over the 11 full years of available data, the ARGO buoys show warming at a rate equivalent to only 0.023 K decade^−1^ (Fig. 5). The buoys did not begin supplying global data till 2004, so that they did not cover the first four of the 11 years mentioned in [37].

The ARGO data (albeit subject to substantial coverage uncertainties in that each buoy must attempt to represent the temperature changes in 200,000 km^3^ of ocean) indeed demonstrate warming of the ocean over the 11-year period for which data are available. There are also measurement uncertainties: Sampling errors of up to 2 K were reported in [59]. Warming trend over the decade was 0.02 K. If this trend were the consequence of 0.5 W m^−2^ of radiative forcing, then the implicit transient-sensitivity parameter would be 0.04 K W m^−2^ compared with the zero-feedback value 0.31 W m^−2^. Even after allowing for thermal inertia in the oceans, the implication is that strongly net-negative temperature feedbacks are in operation, confirming, contrary to an assertion in [37], that little error arises from assuming *r*_*t*_ = 1.

### Values of the transience fraction *r*_*t*_

In [37], it is argued that the choice of *r*_*t*_ = 1 in [6] was “equivalent to an instantaneous response”, but that “this is only true if the heat capacity of Earth is zero”. Here, the authors of [37] have misunderstood the definition of the transience fraction. In [6], the transience fraction was defined as “the fraction of equilibrium sensitivity expected to be attained over *t* years,” but, in the discussion of this variable, it was made plain that its purpose was to allow for nonlinearities specifically arising from the action of temperature feedbacks over different timescales. Thus, where feedbacks are net zero, the instantaneous and equilibrium sensitivity parameters are equal and the transience fraction is accordingly unity. It may, however, be set to any desired lesser value to simulate response lags caused by thermal inertia.

### Reasons why the general-circulation models’ predictions have proven excessive

In [37], it is stated that “temperature trends since approximately 1998 have been at the low end of the CMIP5 distribution”. In fact, it is not appropriate to consider only the period since 1998, for nearly all of that period falls within the current negative phase of the Pacific Decadal Oscillation [60], whose 30-year cycles of positive or warming phases followed by 30-year cycles of cooling phases have had a visible effect on the evolution of global temperature since the beginning of the twentieth century. These cycles must be allowed for in attribution of temperature trends. The simplest method is to ensure that any period of study is either a multiple of 60 years or, if less than 60 years, is centered on a phase transition between the PDO phases. Conveniently, IPCC made its first temperature predictions in 1990 and almost half of the period since then was in a positive PDO phase, the remainder being in a negative phase. As Fig. 1 of [6] demonstrated, IPCC’s central medium-term global-warming prediction in 1990 has proven to be double the observed warming rate, which falls below IPCC’s entire predicted warming interval.

**Fig. 6:**
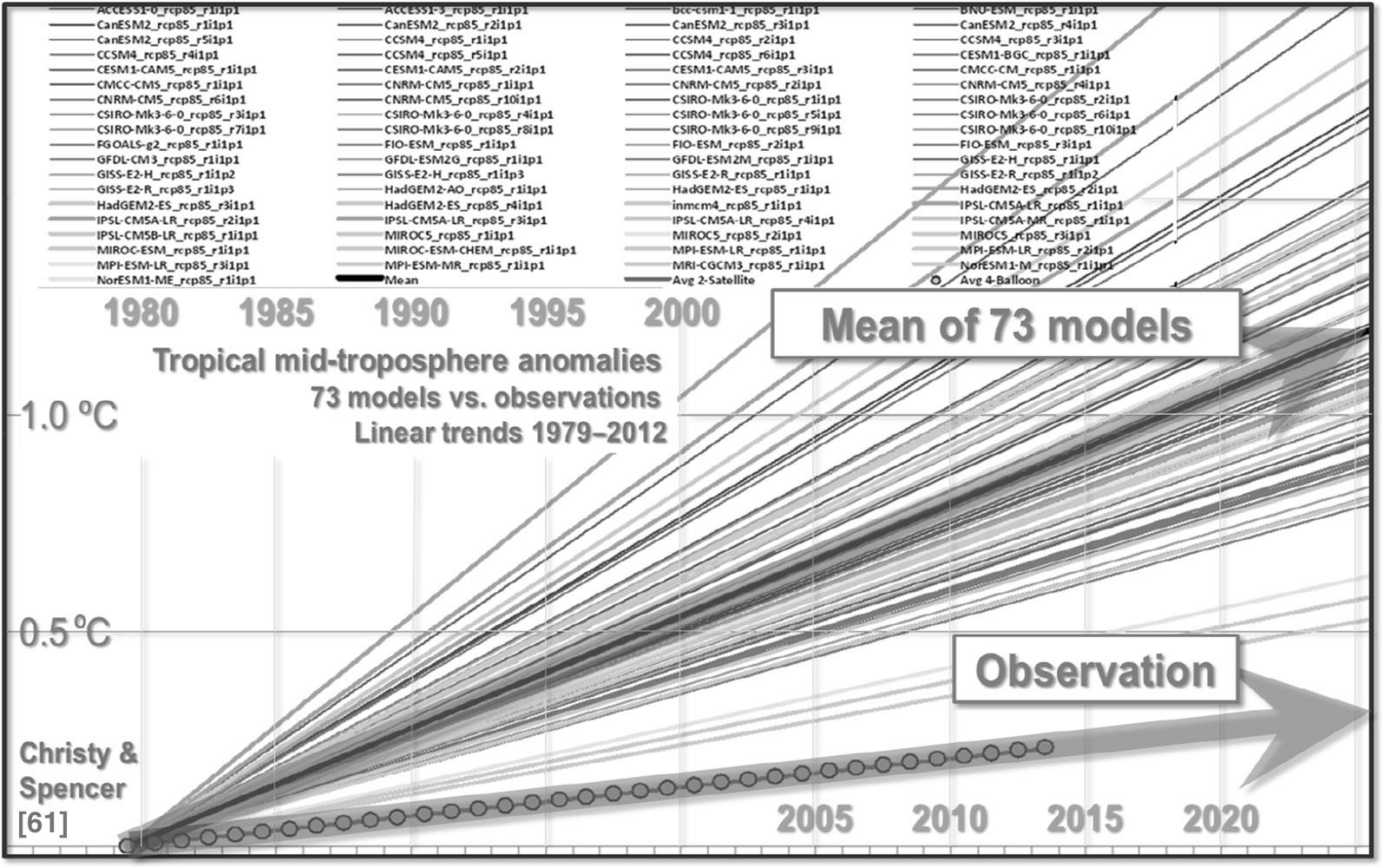
Linear trends on tropical mid-troposphere temperature anomalies projected by 73 models and measured by two coincident observational datasets [61, 62] 1979–2012

A measure of the severity of the models’ failure is shown in Fig. 6, where all linear trends on the influential tropical mid-troposphere temperatures predicted by 73 models exceed those on two global-temperature datasets.

**Fig. 7:**
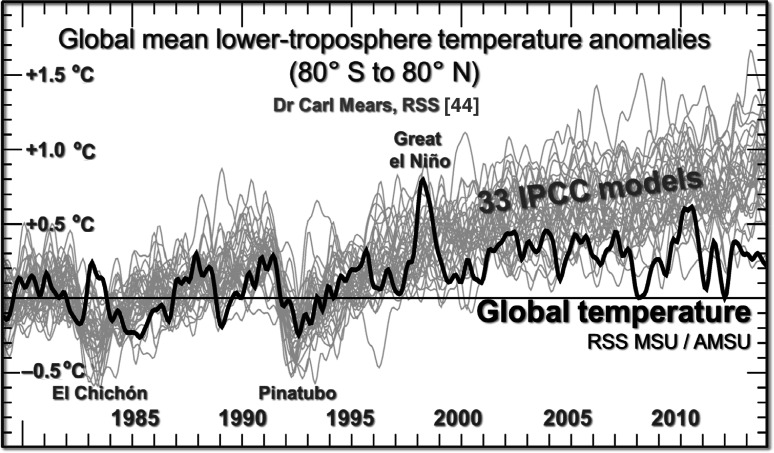
Mean RSS [43] lower-troposphere temperature anomalies, 80°S–80°N, 1979–2014 (black spline-curve) against anomalies predicted by 33 models. Based on a graph by C. Mears [44]

Likewise, RSS [43] indicates a considerable exaggeration of the warming rate by the models (Fig. 7).

**Fig. 8:**
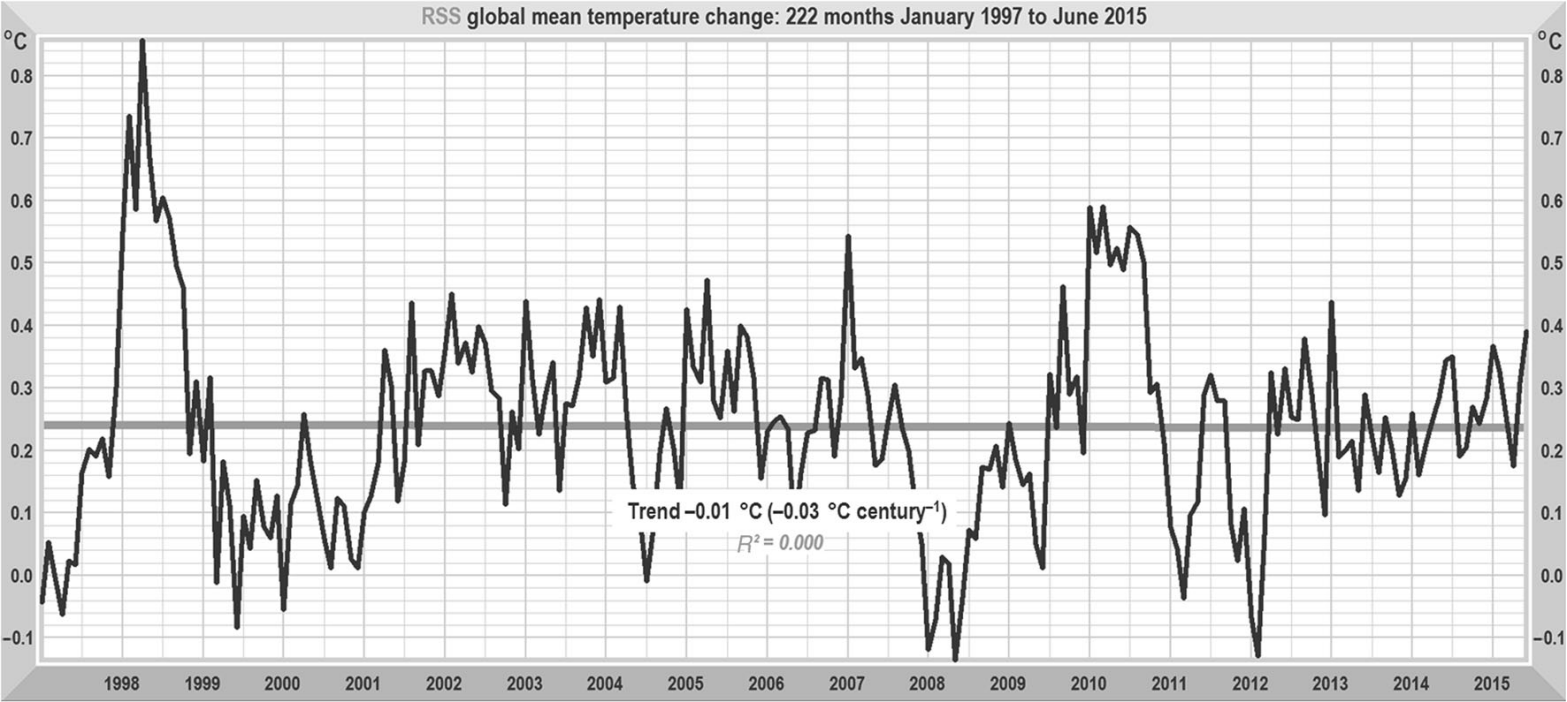
RSS [43] monthly global mean lower-troposphere temperature anomalies, January 1997 to June 2015. Over these 18 years 6 months, the linear trend on the anomalies is approximately zero, notwithstanding CO_2_ concentration rising at a rate equivalent to >200 μmol mol^−1^ century^−1^. One-third of all anthropogenic emissions since 1750 arose in these 222 months

It is recognized in [37] that the general-circulation models have failed in the task of predicting global-temperature change. Indeed, three possible explanations are offered: overestimated radiative forcing, inadequate representation of internal variability and overestimated climate response. It is suggested that the authors of [6] favor the third explanation of the models’ running hot. However, the state of knowledge of the magnitude of radiative forcings and of the mechanisms of internal climate variability is insufficient to allow any definite conclusion to be drawn. As an instance, the startling abruptness and magnitude of the Great El Niño of 1997–1998 (Fig. 8) and its two predecessors over the past 300 years are not yet satisfactorily explained. Accordingly, [6] concentrated on five specific aspects of the models’ representation of climate that were likely to have contributed significantly to the considerable overstatements of warming in the models’ projections.

The second satellite dataset, UAH, shows a similar period of temperature stasis. None of the complex models predicted anything like so long a hiatus in global warming. The discrepancy between prediction and observation over that 18-year period is smaller for the simple model than for the general-circulation models.

### IPCC’s estimates of temperature feedbacks

It is argued in [37] that [6] had misinterpreted IPCC feedback estimates, in that the magnitudes of the principal climate-related feedbacks had been determined by different methods in AR4 and AR5, the latter having included representations of ocean changes and also having assumed a linear feedback response, and in that a second panel in Fig. 9.43 of AR5 had not been reproduced alongside the first.

Here, the authors of [37] are more than somewhat disingenuous. Figure 9.43 of AR5 was stated and intended to provide a direct comparison between the magnitudes of temperature feedbacks in AR4 and AR5. The feedback sum in AR5 is substantially less than that in AR4, because the more complete representation of the coupled ocean-atmosphere system in AR5 has constrained the feedback values (which, however, remain substantially overstated). Furthermore, the second panel of Fig. 9.43 was omitted in [6] because it did not concern itself with individual feedback values; nor did it compare the feedback sums in AR4 and AR5.

For the reasons explained in [6], the feedback values in AR4 lead to the climate sensitivities found in AR4, but the same is not the case for AR5, where equilibrium sensitivity should have been—but was not—reduced by one-third to take account of the reduction in feedback values. Contrary to an assertion in [37], neither the text of AR5 nor the missing panel from Fig. 9.43 in any way explains the reasons why a substantial reduction in feedback values in response to an unchanged forcing might somehow lead to an unchanged climate sensitivity.

As for the fact that AR5 assumed a linear feedback response, the implications of this assumption for climate sensitivity are by no means clear, not least because the values of individual feedbacks, as well as the curves of their evolution over time, are so uncertain. The Appendix to [6], which was lost for reasons of space, is annexed here to outline the mathematical treatment of nonlinear feedbacks.

## Discussion

In [6], the simple model that provided a framework by which some of the assumptions and methods in the general-circulation models could be tested, and five defects in those models were identified and discussed. In [37], three of the five principal findings in [6] were challenged, but, for the reasons described above, the challenges were not compelling. Two of the five conclusions in [6] were not challenged in [37]: that there is no global warming “in the pipeline” in consequence of our past emissions and that the extreme RCP 8.5 scenario is unjustifiable.

In [37], an attempt was made to demonstrate that the predictive skill of the simple model presented in [6] was worse than that of the general-circulation models. However, that attempt merely served to confirm the skill of the simple model in replicating IPCC’s climate sensitivities if IPCC’s values for the model’s parameters were adopted. And tests of the model against observed temperature change show that since 1750 the simple model performs much as the general-circulation models perform and since 1990 the model has performed with very much greater skill than the general-circulation models.

Very nearly all of the attempted criticisms of [6], though presented as criticisms of the simple model, were in fact directed solely at the choice of parameter values, and not of the simple model itself, which the authors of [37] inadvertently validated in accordance with their own methodology by adopting parameter values consistent with IPCC reports and consequently obtaining IPCC estimates of climate sensitivity from the simple model.

One point that emerges from comparison of the relative merits and skills of the simple and general-circulation models is the sheer magnitude of the influence of uncertainty on any attempt to project future climate states. IPCC, to its credit, has consistently conceded that uncertainties in cloud and aerosol forcings and feedbacks are substantial. The recent reevaluation of the impact of anthropogenic aerosols on the determination of climate sensitivity in [41] is a case in point. FAR had made little allowance for aerosols. Subsequent IPCC *Assessment Reports* made so much allowance for them that forcings since the industrial revolution were all but halved. This decision had the effect of greatly increasing estimates of climate sensitivity derived from observation of temperature change since 1750. If [41] is correct, however (the literature is strongly divided on the aerosol question), then perhaps FAR was right all along about the magnitude of post-1750 forcings.

IPCC has also recognized, correctly, that the climate behaves as a coupled, nonlinear, chaotic object and that, accordingly, the long-term prediction of future climate states is not possible [TAR, §14.2.2.2; cf. 63, 64].

The greatest source of uncertainty in determining climate sensitivity lies in the temperature feedbacks. That feedbacks exist is self-evident. However, their magnitudes and even in some instances their signs are unknown. Here, IPCC and the general-circulation models are at fault in assuming that the magnitudes of most temperature feedbacks are well constrained. Plainly, this is not the case, as the reduction in the feedback sum *f* from 2.0 W m^−2^ K^−1^ in AR4 to 1.5 in AR5 demonstrates. For the reasons discussed in [6], it is likely that the feedback sum is net negative, whereupon climate sensitivity cannot exceed an upper bound of 1.3 K per CO_2_ doubling.

The atmosphere, sandwiched between two vast heat sinks, the ocean below and outer space above, has proven unsurprisingly thermostatic during the stable geological conditions of the past 810,000 years [52]. One might only maintain that climate sensitivity was high by asserting—incorrectly, and counter to evidence in [51, 53, 54]—that no large forcings occurred in the later Pleistocene and Holocene and that, therefore, the small temperature change in the past 810,000 years was attributable to large feedbacks operating on small forcings.

Finally, though it is falsely claimed in [37] that in [6] the cited references [16, 19] have been “collectively rebutted”, no specific arguments or references were provided. Such references, in fact, have not been rebutted but rather have become accepted by mainstream science. For example, at least two recent publications [65, 66] suggest findings consistent with the infrared iris hypothesis advanced in [67] by the lead author of [16].

## Conclusions

The general-circulation models now face a crisis of credibility. Not one of them predicted a stasis of as long as 18 years 6 months (Fig. 8) in global temperatures. Indeed, it is often stated that periods ≥15 years without warming are inconsistent with models’ predictions. For instance, [68, 69] state: “The simulations rule out (at the 95 % level) zero trends for intervals of 15 year or more, suggesting that an observed absence of warming of this duration is needed to create a discrepancy with the expected present-day warming rate”. See also [70–72].

The models relied upon in FAR predicted twice as much warming from 1990 to 2014 as has been observed. All models predicted a warming rate in the crucial tropical mid-troposphere considerably in excess of observation. It is no longer credible to ignore these ever-widening discrepancies between prediction and observation. IPCC itself has recognized that, at least as far as medium-term prediction is concerned, the models have failed, raising the legitimate question whether the longer-term predictions may also have been exaggerated, perhaps as greatly as the medium-term predictions.

The best estimate in FAR was that in the 35 years 1990–2025 global temperature would rise at an approximately linear rate by 1.0 K. After 25 of those 35 years, RSS data show just 0.27 K global warming has occurred—a rate equivalent to just 0.11 K decade^−1^. To reach 1.0 K in the next 10 years, the warming rate would have to accelerate to 0.74 K decade^−1^, or almost seven times the rate observed over the past 25 years. Recently, [72] drew attention to the fact that internal variability may play an important role in explaining the apparent near-zero trend in September Arctic sea-ice extent record from 2007 to 2013.

**Fig. 9:**
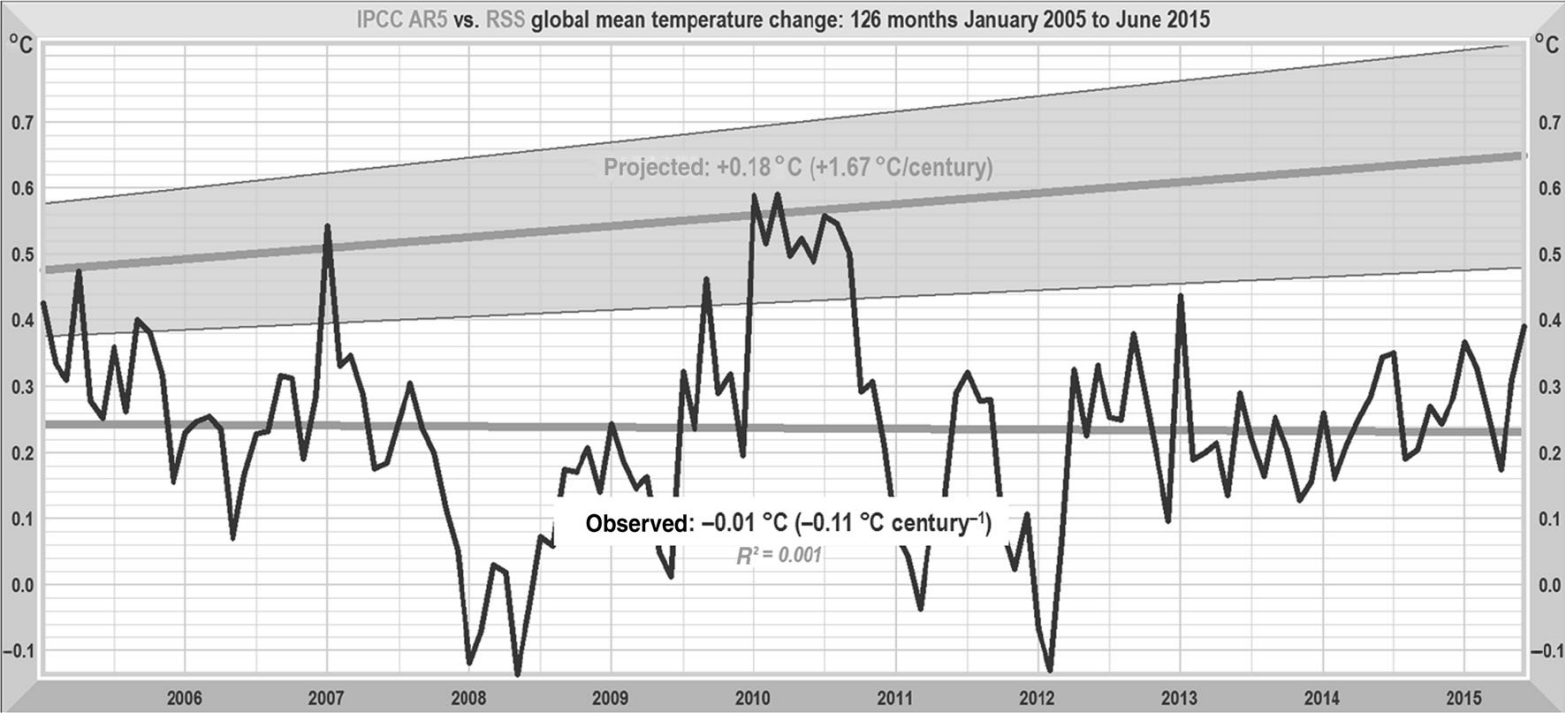
Medium-term AR5 warming projections (gray region) against the RSS [43] monthly global mean lower-troposphere temperature anomalies, January 2005–June 2015, and the trend thereon

IPCC, unlike the authors of [37], has taken note of the failure of the general-circulation models’ predictions and has not sought to maintain that they were accurate when they were not. Figure 9 shows how strongly excessive AR4’s predictions remain. In AR5, IPCC cut its central prediction of medium-term warming from 0.29 to 0.17 K decade^−1^, still well above the observed rate of 0.11 K decade^−1^ over the past 25 years.

Over the past decade, the overprediction even on the new, very much lower rate of predicted medium-term warming has proven to be 0.14 K. The measured atmospheric warming over the period was equivalent to 0.25 K century^−1^, consistent with the underlying ocean warming rate of 0.23 K century^−1^ shown in the ARGO monthly anomalies over a very similar period (Fig. 5).

In the circumstances, it cannot be safely said that, at least as to transient climate sensitivity, the general-circulation models are better validated than the simple model. See [73–75] for a fuller discussion of model validation, which is a more complex task than simply the analysis of RMSE and bias mentioned in [73].

The value of the simple model, particularly when informed by parameters arguably more reasonable than those adopted by the IPCC, is in facilitating examination of the reasons why extreme overpredictions are being made—predictions that governments are acting upon.

As to whether the predictions of general-circulation models or of the simple model will prove correct in the long run, only time will tell. For recent decades, though, the simple model is proving closer to reality.

The pressing question arises why the general-circulation models’ central longer-term projections suggest as much as 3 K warming by 2100 compared with today, let alone why still more extreme estimates of up to 6 K twenty-first-century warming are still made in some circles. The simple model suggests high sensitivity is implausible.

## Electronic supplementary material

Supplementary material 1 (DOCX 23 kb)
