# Supplementary material for: Keeping it simple: the value of an irreducibly simple climate model
Source: Sci Bull (Beijing). 2015 Aug 6;60(15):1378–90. doi: 10.1007/s11434-015-0856-2 (PMC4534509; doi:10.1007/s11434-015-0856-2)
Supplement: Supplementary file 1 — Supplementary material 1 (DOCX 23 kb) [file 11434_2015_856_MOESM1_ESM.docx]

**Appendix 1: Further development of the model**

The simple model may readily be further developed to increase its sophistication, though such developments are beyond the scope of the present paper. For instance, an additional factor might be included in (1) to represent any desired contribution from anthropogenic forcings.

The model might also be made one-dimensional, by representing the latitude. One-dimensional energy-balance models (see [76] for an overview), originally developed by [77-78] and extended by [79-80], have been widely used to introduce students to climate modeling and to examine some peculiarities of the climate system. Some of the more interesting issues that appear when latitude is taken into account are polar amplification of sensitivity to a forcing, the snowball/snow-free bi-stability [76] and the small ice-cap instabilities [81] that arise from the positive ice-albedo feedback.

A one-dimensional model starts with (1) and, at each latitude *φ*, expresses the albedo *α* of the Earth and its clouds, its effective temperature *T_E_*, and the distribution of solar irradiance *S* as functions of *x =* sin *φ*. The one-dimensional model also implicitly assumes that the northern and southern hemispheres are reflections of one another with no net heat flux across the equator. To resolve the latitudinal dimension, the model may be grid-based (as in [79-80]) or based on Legendre polynomials (as in [82]).

The model, however formulated, requires a further equation to describe the poleward transfer of heat. This energy-flux divergence *D* is proportional to –$\nabla$^2^*T*. By Fick’s Law of Diffusion (A1.1), it is expressed by

$D=-\frac{d}{dx}D\left( x \right)\left( 1-x^{2} \right)\frac{dT(x)}{dx}$, | *x =* sin *φ* , (A1.1)

where the diffusion coefficient, $D\left( x \right)$, representing the poleward transfer of energy via oceanic and atmospheric advection, is a tunable parameter that yields a realistic equator-to-pole temperature gradient and can be simplified to render it independent of latitude, with a customary value ~0.65 W m^–2^ K^–1^. In [83] a diffusion coefficient is suggested that is dependent on *x^2^* (consistent with the diffusion coefficient in [77]) so that tropically-averaged motions are better described,

$D\left( x \right)=D_{0}+D_{2}P_{2}\left( x \right)=D_{0}+\frac{1}{2}D_{2}\left( 3x^{2}-1 \right)$, (A1.2)

where $P_{2}(x)$ is the second Legendre polynomial and $D_{0}$, $D_{2}$ are tunable parameters [see also 84]. Use of the second Legendre polynomial is fortunate in that $D\left( x \right)$ should decrease toward the pole, as it does in (A1.2), and the poleward heat flux vanishes at the Equator.

Given the complicated motions of the atmosphere and ocean that transport the energy poleward, such diffusive approximations are conceptually appealing but may not be entirely physically-based [82]. Formulation of diffusion using (8) introduces a term that varies as a function of the equator-to-pole temperature distribution which, necessarily, will alter the temperature response Δ*T_t_* to anthropogenic radiative forcings, thereby changing the response in (1). Specifically, addition of latitudinal diffusion will affect not only the transience fraction, *r_t_*, since the impact of diffusive heat transport and its response to Δ*T* will change the response time to anthropogenic forcing, but also the equilibrium climate-sensitivity parameter, *λ*_∞_.

The model may also be developed to represent non-linear temperature feedbacks. Where feedbacks are non-linear (see [49] for the derivation), (4) becomes (A1.3):

$G_{t}=\left( 1-g_{t}-\frac{{\Delta T}_{t}}{2}\left( \frac{{dg}_{t}}{{dT}_{t}}+\frac{1-g_{t}}{\lambda_{0}}\frac{d\lambda_{0}}{dT} \right) \right)^{-1}$. (A1.3)

In the general case, therefore, the linear-feedback system-gain relation *G_t_ =* (1 – *g_t_*) ^–1^ becomes (A1.4):

$G_{t}={(1-g_{t}-\xi)}^{-1}$ | *ξ* = 0 where feedbacks are linear. (A1.4)

1. Bódai T, Lucarini V, Lunkeit F et al (2014) Global instability in the Ghil-Sellers model. Clim Dyn. doi:10.1007/s00382-014-2206-5
2. Budyko MI (1969) The effect of solar radiation variations on the climate of the Earth. Tellus 21:611–619
3. Sellers WD (1969) A global climatic model based on the energy balance of the earth-atmosphere system. J Appl Meteorol 8:392–400
4. North GR (1975) Theory of energy-balance climate models. J Atmos Sc. 32:2033–2043
5. Ghil M (1976) Climate stability for a Sellers-type model. J Atmos Sci 33:3–20
6. North GR (1984) The small ice cap instability in diffusive climate models. J Atmos Sci 41:3390–3395
7. North GR, Cahalan RF, Coakley JA Jr (1981) Energy balance climate models. Rev Geophys Space Phys 19:91–121
8. Lindzen RS, Farrell B (1977) Some realistic modifications of simple climate models. J Atmos Sci 34:1487–1501
9. Schneider EK, Lindzen RS (1977) Axially symmetric steady-state models of the basic state for instability and climate studies, Part I, Linearized calculations. J Atmos Sci 34:263–279
